# Antioxidants-Related Superoxide Dismutase (*SOD*), Catalase (*CAT*), Glutathione Peroxidase (*GPX*), Glutathione-S-Transferase (*GST*), and Nitric Oxide Synthase (*NOS*) Gene Variants Analysis in an Obese Population: A Preliminary Case-Control Study

**DOI:** 10.3390/antiox10040595

**Published:** 2021-04-13

**Authors:** Amani M. T. Gusti, Safaa Y. Qusti, Eida M. Alshammari, Eman A. Toraih, Manal S. Fawzy

**Affiliations:** 1 Department of Biochemistry, Faculty of Science, King Abdulaziz University, Jeddah 21589, Saudi Arabia; a_gusti2010@hotmail.com (A.M.T.G.); squsti@kau.edu.sa (S.Y.Q.); 2 Department of Medical Laboratory, Biochemistry, King Fahad Armed Forces Hospital, Jeddah 21159, Saudi Arabia; 3 Department of Chemistry, College of Science, University of Ha’il, Ha’il 2440, Saudi Arabia; eida.alshammari@uoh.edu.sa; 4 Department of Surgery, School of Medicine, Tulane University, New Orleans, LA 70112, USA; etoraih@tulane.edu; 5 Department of Histology and Cell Biology (Genetics Unit), Faculty of Medicine, Suez Canal University, Ismailia 41522, Egypt; 6 Department of Medical Biochemistry and Molecular Biology, Faculty of Medicine, Suez Canal University, Ismailia 41522, Egypt; 7 Department of Biochemistry, Faculty of Medicine, Northern Border University, Arar 1321, Saudi Arabia

**Keywords:** antioxidants-related genes, CAT, GPX, GST, NOS, SOD, obesity, single nucleotide polymorphism

## Abstract

Oxidative stress and antioxidants play an important role in obesity etiopathology. Genetic variants, including single nucleotide polymorphisms (SNPs) of the antioxidant-related genes, may impact disease risk in several populations. This preliminary study aimed to explore the association of 12 SNPs related to superoxide dismutase (*SOD*), catalase (*CAT*), glutathione peroxidase (*GPX*), glutathione-S-transferase (*GST*), and nitric oxide synthase (*NOS*) genes with obesity susceptibility in a Saudi population. A total of 384 unrelated participants, including 154 (40.1%) obese individuals, were enrolled. TaqMan OpenArray Genotyping assays were used. Six SNPs were significantly more prevalent in obese cohorts: (1) *GSTM1* rs1056806*C/T; (2) *SOD1* rs2234694*A; (3) *SOD2* rs4880*G; (4) *SOD3* rs2536512*A; (5) *GPX1* rs1800668*A; (6) *NOS3* rs1799983*G. Four SNPs were associated with higher obesity risk under heterozygote and dominant models for *GSTM1* rs1056806 (C/T), homozygote model for *SOD2* rs4880 (A/G), and homozygote and recessive models for *GPX1* rs1800668 (A/G). In contrast, *SOD3* rs2536512 (A/G) were less likely to be obese under heterozygote and dominant models. The CGAG, CAAA, TGGG, and CGAG combined genotypes showed a higher risk of obesity. In conclusion, the present results suggest that oxidative-stress-related genetic determinants could significantly associate with obesity risk in the study population.

## 1. Introduction

Obesity and overweight have grown to epidemic proportions resulting in more than 4 million individuals dying/year in 2017, according to the Global Burden of Disease Study [1]. The prevalence of these disorders continues to rise in developing and developed countries representing a growing public health problem of concern [2]. Accumulating evidence demonstrated that high body mass index (BMI) is a major risk factor for the disability-adjusted life years in the Saudi population due to the recent demographic changes with longer life expectancy and lifestyle changes resulting from rapid urbanization and industrialization [3,4,5,6].

A constellation of risk factors, including environmental and genetic ones, has been implicated in obesity etiopathology [7]. One of these factors is the oxidative stress that can induce obesity and the related comorbidities by promoting white adipose tissue deposition and food intake alteration [8]. A significant direct correlation has been observed between oxidative stress biomarkers and BMI [9]. Several in vitro studies reported that increased oxidative stress and the reactive oxygen species could augment adipocyte proliferation, differentiation, and growth [10,11,12] and control hunger and satiety behaviors [13]. Interestingly, there is a mutual relation between oxidative stress and obesity, as abnormal fat accumulation can elicit a pro-inflammatory and pro-oxidant state through various biochemical and cellular mechanisms [14,15]. 

The glutathione S-transferases (GSTs), which detoxify the endogenously formed electrophilic compounds, including the lipid peroxidation products, showed a white adipose tissue-specific downregulation [16]. Additionally, the antioxidant enzyme activities of glutathione peroxidase (GPx) and superoxide dismutase (SOD) were reported to be dysregulated in red blood cells and serum of obese individuals compared to controls [17,18,19,20,21,22]. Moreover, the activities of Cu-Zn and Mn-dependent SOD, GPx, and catalase (CAT) were significantly downregulated in the abdominal adipose tissue of ovariectomized metabolic syndrome female rat model (to mimic the postmenopausal status) when compared to metabolic syndrome group without receiving treatment or the group received external estrogens [23]. Dysregulated nitric oxide synthase (NOS) enzyme family expression was evident at the mRNA and protein levels in isolated fat cells and adipose tissue sections derived from obese male subjects versus non-obese individuals [24,25,26]. 

Several single nucleotide polymorphisms (SNPs) related to the antioxidant enzymes can impair enzyme activities with subsequent accumulation of reactive oxygen species associated with various diseases/disorders, including obesity [27,28,29].

The GSTs comprise three enzyme families, the most investigated being the cytosolic ones, which include seven classes, Alpha, Mu, Pi, Sigma, Theta, Omega, and Zeta, each encoded by a separate gene [16]. The *GST* Mu 1 (*GSTM1*) rs1056806 [C/T], *GST* theta 1 (*GSTT1*) rs17856199 [A/C], *GST* Pi 1 (*GSTP1*) rs1695 [A/G], and microsomal *GST3* (*mGST3*) rs2065942 [C/T] variants of this family can impair the GSTs catalytic activities and are studied in multiple diseases, including obesity and related comorbidities [16,27].

The *SOD* gene family members are located on different chromosomes, “*SOD1* on 21q22.11, *SOD2* on 6q25.3, and *SOD3* on 4p15.3–p15.1” are coding for the “intracellular CuZn-SOD (SOD1), mitochondrial Mn-SOD (SOD2), and extracellular EC-SOD (SOD3)” enzymes, respectively [30,31]. The *SOD1* rs2234694 [A/C] is located at the third exon/intron splicing site, the *SOD2* rs4880 [A/G] has been found to impact the “mitochondrial targeting peptide; MTP” domain of the enzyme [32], and the *SOD3* rs2536512 [A/G] results in alanine substituted by threonine; collectively, they have been associated previously with obesity risk [29,33]. The promotor *CAT* rs7943316 [A/T] SNP (−21A/T) was associated with increased body fat (%), including the visceral one in the obese central Mexico population [34]. While the *GPX* gene cluster includes eight types (*GPX*1-8), the *GPX1* and *GPX4* variants were associated previously with obesity or related comorbidities [16,35]. The *GPX1* mapped to chromosome 3p21.3 contains the polymorphism rs1800668 [G/A], which is likely to affect the encoded cytoplasmic enzyme’s activity [36]. Although the *GPX4* rs713041 [C/T] variant, located in the 3’UTR, the mRNA region important for selenocysteine insertion, was not investigated with obesity, this polymorphism showed risk modulation for diabetic retinopathy and cardiovascular autonomic neuropathy in patients with type 1 diabetes [37,38].

Lastly, the NOS family consists of three members; endothelial NOS (eNOS/NOS3) reported to have an anti-obesogenic effect, the inducible NOS (iNOS/NOS2) that promotes insulin resistance, and the neuronal NOS (nNOS/NOS1) that appears to act as appetite regulator [39]. These three isozymes can impact obesity etiology through NO production that plays essential roles in regulating adiposity, energy expenditure, and insulin sensitivity [39,40]. The *NOS2* rs2297518 [A/G] and *NOS3* rs1799983 [G/T] genetic variations were associated with insulin resistance, obesity, and/or a higher BMI in several populations [41,42,43]. 

As the results of most genetic studies related to the polymorphisms mentioned above were inconsistent and mainly carried out in patients of European descent, the authors were interested in exploring the potential genetic association of the selected 12 SNPs with obesity in a sample of the Middle Eastern population and relating the different genotypes with the available clinic-laboratory data.

## 2. Materials and Methods

### 2.1. Subjects 

This case-control study enrolled a total of 384 adult unrelated Saudi individuals; 143 women and 241 men accounting for 37.2 and 62.8%, respectively. These subjects were recruited from Endocrinology and Diabetes center outpatient clinic, King Fahd Armed Forces Hospital (KFAFH), Jeddah, KSA during their routine check-up program. Subjects with complicated or severe diseases (e.g., stroke, diabetic complications, cardiac diseases, renal/hepatic disorders, psychiatric disorders, cancers) were excluded. Informed consent was obtained from all subjects before participating in the study. All the participants underwent a complete medical history and physical examinations. Anthropometric parameters, including weight and height, and arterial blood pressure (BP), were measured. Body mass index (BMI) was calculated “as weight (kg) divided by the square of height (m)”, and accordingly, obesity was defined from a BMI of ≥30 kg/m^2^ [4]. Hypertension was defined as stated previously [44]. Ethical approval was obtained from the institutional review board at the King Fahd Armed Forces Hospital (approval No. 201_19/04/2017), Jeddah, KSA.

### 2.2. Sample Collection and Laboratory Analysis

A total of ten milliliter peripheral blood samples were collected after an overnight fast (10–12 h) on two ethylenediaminetetraacetic acid tubes and one serum separator tube. The former tubes were sent for either the molecular analysis (4 mL) or glycated hemoglobin A1c (HbA1c; 3 mL) determination (VARIANT II TURBO Hb Testing System, Bio-Rad, Hercules, CA, USA). The last tube (3 mL) was centrifuged immediately at 3000 rpm × 12 min to separate the serum for other biochemical assays measurements, including serum glucose and lipid profile [total cholesterol (TC), high-density lipoprotein-cholesterol (HDL-C), and triacylglycerol (TG)] (Cobas c701, Roche Diagnostics, Indianapolis, IN, USA). As serum TG levels in all included samples were less than 4.5 mmol/L, the low-density lipoprotein-cholesterol (LDL-C) levels were calculated according to the Friedewald equation [45]. All abnormal lipid and lipoprotein profiles were justified by the National Cholesterol Education Program and Adult Treatment Panel III (NCEP-ATP III) guidelines [46]. A value less than the 50th percentile of HDL-C (i.e., <1.0 mmol/L) is considered a low level. Hypertriglyceridemia was specified at a fasting serum TG level of ≥1.7 mmol/L. Participants with dyslipidemia were defined as having an abnormal level of one or more lipid profiles, currently on lipid-lowering drugs, or having a history of a lipid disorder. Serum insulin levels were measured by Electrochemi-luminescence Immunoassay (Cobas e 602 immunoassay analyzer, Roche Diagnostics, Indianapolis, IN, USA). 

### 2.3. SNP Selection and Genotyping

Based on (1) a dbSNP (www.ncbi.nlm.nih.gov; accessed on 28 October 2019) search for the SNP minor allele frequency (MAF) > 0.05 and (2) the previous literature that showed evidence of functional significance and association of the specified SNPs with obesity risk in various populations, the authors selected 12 variants related to *GSTM1*, *GSTT1*, *GSTP1*, *MGST3*, SOD1, *SOD2*, *SOD3*, *CAT*, *GPX1*, *GPX4*, *NOS3*, and *NOS2* genes (Table S1). Genomic DNA was extracted from peripheral blood leukocytes using Roche MagNA Pure Compact Nucleic Acid-based Isolation Kit I (Catalog no. 03730964001; Roche Diagnostics GmbH, Mannheim, Germany) according to the manufacturer’s instructions. Nanodrop 2000/2000c Spectrophotometer V1.0 (Thermo Fisher Scientific, Wilmington, DE, USA) and gel electrophoresis were applied to evaluate the concentration and integrity of the extracted DNA, respectively. Amplification and allelic discrimination were performed in a GenaTi research center (King Abdulaziz University, King Fahad Medical Research Center) using Quant Studio 12K Flex Real-Time PCR System (Applied Biosystems, Foster City, CA, USA). Five microliters TaqMan GTXpress Master Mix (2×) (Cat no. 4403311, Applied Biosystems) were mixed with 0.5 μL TaqMan genotyping assay mix (20×), 2.5 μL nuclease-free water, and 2.0 μL gDNA (20 ng/μL) in a total reaction volume of 10 μL/sample. The genotyping assays ID and the ready-to-use primer, and probe sequences for the specified study variants are summarized in Table S1. The PCR program was run as follows: enzyme activation at 95 °C for 10 min followed by 40 cycles of denaturation at 95 °C for 15 s and annealing/extension at 60 °C for 1 min [47]. All the quality control measurements were followed in each run, including using the appropriate controls. About 10% of the samples were reassessed with a 100% recall rate supporting the genotyping efficiency. 

### 2.4. Statistical Analysis

Data were analyzed using the R software version 4.0.2 (RStudio 3.0.1) and BM Statistical Package for the Social Sciences (SPSS) Statistics for Windows, version 26.0 (IBM Corp., Armonk, NY, USA). After testing the normality of the continuous variables, using the Shapiro–Wilk test, quantitative data were expressed as means ± standard deviation (normally distributed data) or median and interquartile range, while qualitative data were expressed as numbers and percentages. Two-sided Chi-square, Student-t, and ANOVA tests were used for parametric data, while Mann–Whitney U and Kruskal–Wallis tests were employed for non-parametric variables. Analysis of allele frequencies (number of copies of a specific allele divided by the total number of alleles in the group) and genotype frequencies (the number of each genotype divided by the total number of individuals within the group) was carried out [48]. Genotype frequencies were assessed for deviation from the Hardy–Weinberg equation (HWE) using an online excel sheet to compare observed versus expected values. A Chi-square test was used to check goodness-of-fit [49]. Single and polygenic SNP analyses were performed. Genotype combination analysis was performed to associate obesity using the online SNPStats software (www.snpstats.net, accessed on 18 July 2020) [50]. The relationship between allele frequencies and obesity was determined under different inheritance models with logistic regression analysis after adjustment for age and sex. Genetic association models included heterozygote comparison, homozygote comparison, dominant model, and recessive models [51]. Iteration of analysis was performed to test the association with the risk of obesity. Significant results in univariate analysis were plotted as a forest plot using STAT version 16.0 (StataCorp. 2019. Stata Statistical Software: Release 16. College Station, TX, USA: StataCorp LLC). Next, multivariable regression analysis was performed to include significant molecular markers from univariate analysis with clinical data and laboratory testing. The Hosmer–Lemeshow test was used to assess goodness-of-fit. Results were reported as odds ratio (OR) and 95% confidence interval (CI). Regression models were applied with genetic variants alone then repeated to integrate clinical and laboratory data [51]. Pearson’s correlation test was performed for genotype-genotype and genotype–phenotype correlation. Both clinical and laboratory findings were tested for their association with each polymorphism. The correlation matrix was plotted in R using RColorBrewer, ggpubr, tidyverse, Hmisc, and corrplot packages [52]. A *p*-value of <0.05 was considered statistically significant. 

## 3. Results

### 3.1. Characteristics of Obese and Non-Obese Cohorts

Table 1 shows the clinic-laboratory characteristics of the study subjects. The study population’s mean age was 38.7 ± 15.5 years (ranged 23 to 83 years old). Of these, 154 subjects (40.1%) were obese. Obese cohorts were significantly older (41.49 ± 16.2 vs. 36.96 ± 14.7, *p* = 0.005). There was no significant difference in their sex (*p* = 0.10). However, they were more likely to have hypertension (20.1 vs. 11.7%, *p* = 0.029) and dyslipidemia (22.7 vs. 11.3%, *p* = 0.004). Laboratory testing showed higher levels of serum glucose (*p* = 0.003) and insulin (*p* = 0.006).

### 3.2. Allelic Discrimination Analysis in Obese and Non-Obese Subjects

Table 2 summarizes allele and genotype frequencies of 12 genetic variants in obese and non-obese cohorts. Of these, six SNPs were significantly associated with development of obesity. (1) *GSTM1* rs1056806*C/T heterozygosity was significantly more prevalent in obese cohorts (21 vs. 12%, *p =* 0.039); (2) *SOD1* rs2234694*A allele was exclusively present in patients (100 vs. 97%, *p* = 0.048); (3) *SOD2* rs4880*G allele was more common in obese cohorts (52 vs. 43%, *p* = 0.038); (4) *SOD3* rs2536512*A allele was associated with obesity (56 vs. 51%, *p* < 0.001), similarly, A/A genotype was more frequent in patient group (36 vs. 23%, *p* = 0.033); (5) *GPX1* rs1800668*A allele carriers were more representative in patients (25 vs. 19%, *p* = 0.041); (6) *NOS3* rs1799983*G allele was more prevalent in obese group (83 vs. 75%, *p* = 0.029). 

### 3.3. Monogenic Risk of Obesity with Each Polymorphism

**Figure 1:**
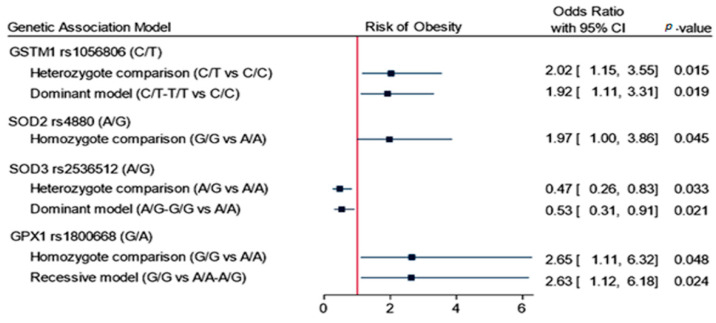
Association of genetics variant panel with the risk of obesity. Data are presented as the odds ratio and 95% confidence interval (CI). *p*-value < 0.05 was considered as statistically significant. Binary logistic regression analysis was adjusted for age and sex.

Inheritance association models revealed that four SNPs to have a prediction value for developing obesity, namely *GSTM1* rs1056806 (C/T), *SOD2* rs4880 (A/G), *SOD3* rs2536512 (A/G), and *GPX1* rs1800668 (A/G). *GSTM1* rs1056806 showed a higher risk of obesity under heterozygote comparison (C/T vs. C/C: OR = 2.02, 95%CI = 1.15–3.55, *p* = 0.015) and dominant models (C/T-T/T vs. C/C: OR = 1.92, 95%CI = 1.11–3.31, *p* = 0.019). *SOD2* rs4880 had higher odds of developing obesity under homozygote comparison model (G/G vs. A/A: OR = 1.97, 95%CI = 1.0–3.86, *p* = 0.045). *GPX1* rs1800668 had an increased risk of obesity under homozygote comparison (G/G vs. A/A: OR = 2.65, 95%CI = 1.11–6.32, *p* = 0.048) and recessive models (G/G vs. A/A-A/G: OR = 2.63, 95%CI = 1.12–6.18, *p* = 0.024). In contrast, *SOD3* rs2536512 carriers were less likely to be obese under heterozygote comparison (A/G vs. A/A: OR = 0.47, 95%CI = 0.26–0.83, *p* = 0.033) and dominant models (A/G-G/G vs. A/A: OR = 0.53, 95%CI = 0.31–0.91, *p* = 0.021) (Figure 1 and Table 2).

### 3.4. Polygenic Risk of Obesity in Association with Gene Variants

Genotype combination analysis of *GSTM1* rs1056806 (C/T), *SOD2* rs4880 (A/G), *SOD3* rs2536512 (A/G), and *GPX1* rs1800668 (A/G) revealed four common combinations with higher risk of obesity; CGAG (OR = 6.15, 95%CI = 1.46–25.8, *p* = 0.014), CAAA (OR = 5.0, 95%CI = 1.23–20.3, *p* = 0.025), TGGG (OR = 3.25, 95%CI = 1.06–9.95, *p* = 0.040), and CGAG (OR = 2.95, 95%CI = 1.27–6.86, *p* = 0.012) (Table 3).

### 3.5. Association of Gene Variants with Clinical and Biochemical Characteristics

**Figure 2:**
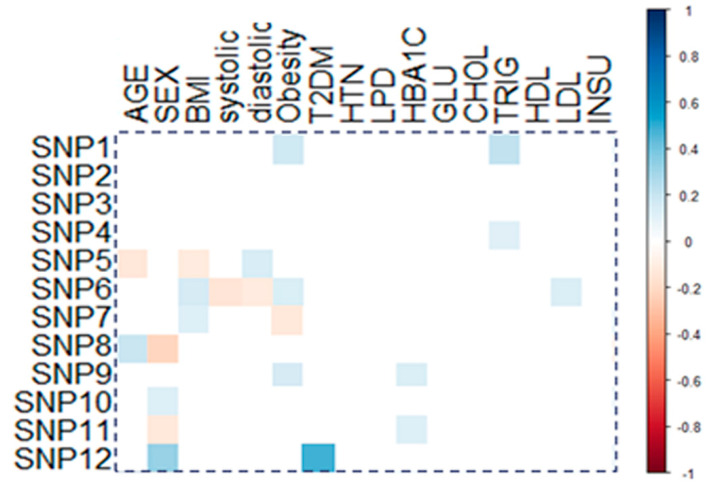
Correlation of genetic variants with patients’ characteristics. Correlation between gene variants and demographic/clinic-laboratory characteristics. Pearson correlation was applied, and a significant correlation coefficient is only shown. BMI: body mass index; systolic: systolic blood pressure, diastolic: diastolic blood pressure; T2DM: type 2 diabetes mellitus, HTN: hypertension; LPD: hyperlipidemia, HbA1C: glycosylated hemoglobin, GLU: glucose; CHOL: total cholesterol, TRIG: triacylglycerol; HDL: high-density lipoprotein; LDLL: low-density lipoprotein; INSU: serum insulin; SNP1: *GSTM1* rs1056806, SNP2: *GSTT1* rs17856199, SNP3: *GSTP1* rs1695, SNP4: *MGST3* rs2065942, SNP5: *SOD1* rs2234694, SNP6: *SOD2* rs4880, SNP7: *SOD3* rs2536512, SNP8: *CAT* rs7943316, SNP9: *GPX1* rs1800668, SNP10: *GPX4* rs713041, SNP11: *NOS3* rs1799983, SNP12: *NOS2* rs2297518.

Correlation analysis of the study gene variants with clinical and laboratory data is shown in Figure 2. Obesity was positively correlated with *GSTM1* (*r* = 0.109, *p* = 0.035) and *SOD2* (*r* = 0.121, *p* = 0.046). Serum LDL-C levels also showed weak positive correlation with *SOD2* (*r* = 0.113, *p* = 0.047), and type 2 diabetes mellitus showed moderate correlation with *NOS2* (*r* = 0.397, *p* < 0.001). 

## 4. Discussion

Oxidative-stress-related indices are reported to be associated with increased obesity risk and related comorbidities [10,15,53]. To the best of our knowledge, no studies have investigated the role of the selected 12 antioxidant-related SNPs collectively in the same obese/non-obese cohort. Preliminarily, the obese group in this study was older, had a higher frequency of dyslipidemia and hypertension, and had higher serum glucose and insulin levels than non-obese individuals. This reflects the typical clinical presentation of obesity and associated comorbidities. Several population surveys have shown that the obesity prevalence increases progressively from 20 to 60 years of age [54], and significant associations were reported between obesity and hyperglycemia [55], dyslipidemia [56], hyperinsulinemia [57], and hypertension [58].

In this study, we found that six SNPs were significantly more prevalent in obese cohorts: (1) *GSTM1* rs1056806*C/T heterozygosity, (2) *SOD1* rs2234694*A allele, (3) *SOD2* rs4880*G allele, (4) *SOD3* rs2536512*A allele, (5) *GPX1* rs1800668*A allele, and (6) NOS3 rs1799983*G allele. Furthermore, four variants from these SNPs showed significant association with the risk of developing obesity under several genetic association models that will be detailed in the next sections. 

**Figure 3:**
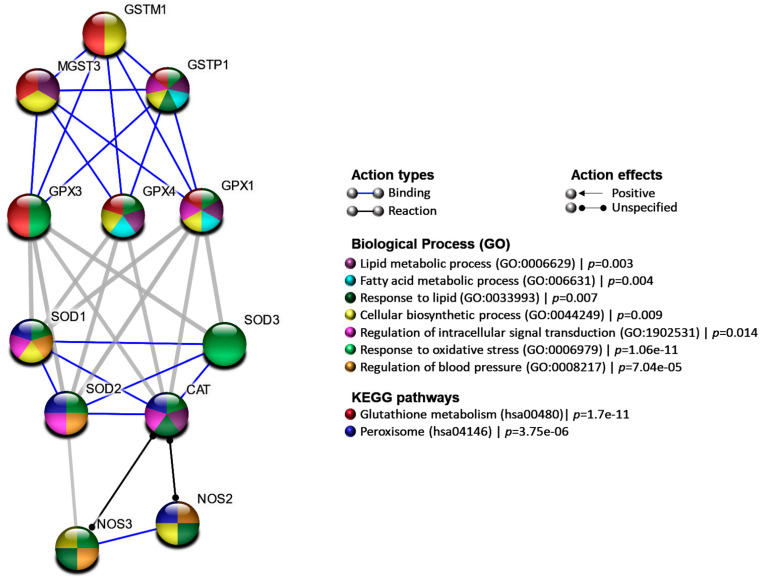
Protein–protein interaction network. Nodes represented the genes colored according to the functional enrichment pathways and biological process, while edges showed interactions. STRING database version 11.0 was used.

Our enrichment analysis of the studied antioxidant enzymes revealed their implication in several obesity-related pathways and processes such as lipid metabolism/response to lipid processes and dysregulation of fatty acid metabolism [59], cellular biosynthetic process derangement [60], intracellular signal transduction regulation, response to oxidative stress [61], and regulation of blood pressure [62], as shown in Figure 3. 

As a member of phase II drug metabolism-related family catalyst, GSTM1 can detoxify the electrophilic products generated from lipid peroxidation that considers one of the major pathogenic cellular changes in obesity onset and progression [63]. Although Yang, [64] did not find an association of *GSTM1* rs1056806 (c.528C > T) polymorphism with susceptibility of obesity in the Korean cohort (117 overweight/obese cases versus 125 non-overweight/obese subjects), a higher frequency of the heterozygous genotype was evident in the current obese subjects than non-obese. Furthermore, this synonymous variant (i.e., associated with no change to the encoded amino acid) showed a significant association with obesity risk under heterozygote comparison and dominant models. Previous estimates identified that 5–10% of human genes include at least one locus where synonymous variants could be harmful and impact protein conformation, expression, and/or function by affecting the post-transcriptional processing [65]. The later report highlighted the importance of including such types of variants in follow-up mechanistic and functional studies. The inconsistency observed between our finding and the previous Korean study may be attributable to the limited sample size, the different genotyping methodology, and different genetic backgrounds among the study population. This warrants work replication in large-scale studies with different ethnic populations to validate the findings. 

The vital family of antioxidant catalysts in humans that neutralize the reactive oxygen species “superoxide anion” consists of three isozymes: Cu, Zn-dependent cytosolic SOD1, manganese-dependent mitochondrial SOD2, and Cu, Zn-dependent extracellular SOD3 [29,31]. The *SOD1* rs2234694*A allele was more prevalent in the current obese cohort relative to non-obese individuals. This result agrees with Lewandowski et al., in which this variant could be associated with differences in SOD concentrations based on the obesity status [33]. A previous study found that patients with diabetes mellitus homozygous for the minor allele were characterized by a decline in the total SOD activity compared to their counterparts [66]. Additionally, *SOD2* rs4880*G allele was more frequent in the current obese cohort than controls, and the GG genotype carriers had two-fold the risk to be obese versus the AA carriers. This finding was consistent with previous studies [33,67].

Interestingly, the activity of the mitochondrial SOD2 isozyme coded by this gene has been underscored in neutralizing the mitochondrial O_2_^•−^, preventing the uncoupler proteins hyperactivity that is induced by this reactive oxygen species in the context of obesity [68]. The rs4880 variant causes alanine to valine substitution, which was reported to be associated with conformational changes in the “mitochondrial targeting peptide” of the enzyme, which alters the importing efficiency and the enzyme catalytic activity in the mitochondrial matrix [69]. This type of substitution has also been associated with variability in SOD total activity, leptin, total cholesterol, and oxidative-stress-related biomarkers blood levels in obese/non-obese individuals [66,70]. This latter association can support the significant correlation this variant showed with LDL-C levels in the study cohort (Figure 2). Furthermore, in the present obese cohort, the GG genotype code for alanine showed increased odds of obesity than other genotype carriers. This means that higher enzyme activity could be associated with obesity risk. Under metabolic derangement, higher SOD2 isozyme activity might induce higher mitochondrial hydrogen peroxide (H_2_O_2_) accumulation associated with oxidative-stress-related insulin resistance, dyslipidemia, and unbalanced energy homeostasis, which characterize the obesity state [71,72]. Interestingly, adipocyte-targeted “*SOD2* knockout mice” has prevented high-fat-diet-induced obesity and insulin resistance [73], supporting the present findings.

Otherwise, *SOD3* rs2536512*A allele carriers in the present cohort were less likely to be obese under heterozygote comparison and dominant models. This variant causes alanine to threonine substitution at position 58 (Ala58Thr) in SOD3 isozyme, and “A-allele” was associated previously with higher SOD activity [74]. This could explain the protective role this allele displays in the present obese cohort. Although, to the authors’ knowledge, no previous study related this variant to the increased/decreased odds of obesity development, other studies have explored the role of this polymorphism with type 2 diabetes mellitus [75] and altered serum TG/HDL-C levels [71]. Originally SOD family was related to obesity due to “its protective role as an antioxidant”, and high SOD3 concentration/activity is expected to neutralize the high levels of the superoxide anion (O_2_^•−^) effectively [29,71]. This isozyme’s availability on the extracellular compartment and the endothelial cells add advantage to its action to neutralize (O_2_^•−^) generated in the extracellular matrix and discontinue the peroxynitrite generation [76]. This latter pro-oxidant was recently implicated in impaired “endothelium-dependent vasodilation”, one of the mediators of “obesity-induced hypertension” [77]. Additionally, supporting our findings, overexpression of *SOD3* in adipose tissues in cases of diet-induced obesity was associated with blocking the development of obesity, fatty liver, and insulin resistance [78]. The latter investigators also found that even in the liver, increased SOD3 activity was associated with upregulation of the genes responsible for “energy expenditure”.

The cytosolic GPx1 enzyme is one of the most studied catalysts of the GPx family. Related variants to this SNP exist in the human population and modify GPx1 activity as reviewed in detail previously [16]. The 5’UTR *GPX1* rs1800668*A allele was more prevalent in our obese cohort than non-obese one and showed association with obesity risk under homozygous comparison and recessive models. Although SNP does not impact the structure of the protein directly, the GG genotype carriers showed relatively higher GPx1 activity than GA or AA genotype carriers [36]. This variant was not associated with obesity risk previously, up to the authors’ knowledge, and it is the least studied antioxidant variant in association with different diseases [79]. However, given the essential roles that the encoded enzyme plays in the reduction in hydroperoxides of non-esterified polyunsaturated fatty acids [35], as well as regulation of “insulin sensitivity” and “obesity-induced insulin resistance” [80,81], it is not surprising that it is one of the obesity-related susceptibility SNPs that deserves work replication in other populations to confirm this finding.

Lastly, the NOS3 enzyme has been identified to play an essential role in lipolysis modulation, and obesity condition per se has profound effects on covalent modification of the NOS enzyme by insulin-dependent activation of protein kinase B [39]. The *NOS3* rs1799983 (Glu298Asp) genetic variant may influence *NOS3* expression and serum nitrate levels [82]. Our study results are consistent with MacKenzie and colleagues’ observation, in which the rs1799983GG genotype was frequently present in obese children, but the association with the disease did not reach statistical significance [83]. So far, various studies in other populations have explored the association between this variant and obesity risk [39,42,84,85]. Malhotra et al. reported that obese rs1799983*T allele carriers exhibited increased cardiac output and decreased NO metabolites during stress than non-obese participants [84]. In another study, the association between this variant and the diabetes risk was modified by BMI, with clear evidence of interactions between obesity and the rs1799983TT genotype [85]. Nasr et al. also observed a significant association of rs1799983*T allele with obesity and BMI in the Tunisian population [42]. Additionally, recently, Pawlik et al. reported a significant association of this variant with BMI and waist circumference in female patients with unstable angina [43]. Although in some studies, including ours, the rs1799983*G allele was more prevalent in the obese cohort than controls that differs from other studies in which the rs1799983*T allele was the risky one, previous reports have indicated interethnic differences in rs1799983 distribution and evidence for linkage disequilibrium with other SNPs exists among populations [86,87].

Apart from the variants discussed with obesity risk, there were no significant differences in the prevalence of other studied SNPs among the current groups. However, some variants showed a significant correlation(s) with the studied cohort’s laboratory parameters and/or associated comorbidities. For example, the *NOS2* rs2297518 variant showed a significant correlation with diabetes mellitus (Figure 2). This missense variant (S608L), which is mapped in the catalytic domain of the enzyme [88], was reported to increase NOS2 activity and impact many disease prevalence [89]. This observation agrees with other genetic association studies on type 2 diabetes mellitus cohort in our lab [90] and in Caucasians [91]. 

Genotype combination analysis for the significantly associated four SNPs with the risk of obesity development in the present study revealed that four common combinations were identified to increase the odds of obesity. These combinations were CGAG (6-fold), CAAA (5-fold), TGGG (3-fold), and CGAG (2-fold). Recent evidence suggests that investigating multiple SNPs that increase susceptibility to obesity may predict the degree of risk for obesity and strategically contribute to weight-management programs [92,93]. Single SNP analysis is often underpowered due to the small effect size of individual SNP; however, the combined or additive effect of several variants from different loci have a larger effect size and, hence, greater predictive power, as evident in recent obesity studies [92,94,95].

Although the present study uncovered the association of antioxidants related to 12 SNPs collectively with obesity risk in a sample of the Middle Eastern population for the first time, having GWAS would offer further insight in future association studies. As a preliminary study, this work needs to be extended and validated on large-scale multicenter studies. The absence of functional studies to explore the studied variants’ impact on the encoded antioxidant enzyme levels/activities and other oxidative-stress-related biomarkers could limit this study. Additionally, given the high consanguinity rate in the Saudi population [96] and unavailability of the data related to this parameter in the present work, the potential effects of consanguinity with other genetic susceptibility variants on obesity risk should be considered. 

Further functional studies with replication of the work in other ethnic populations are recommended to support the “ethnic-specific” genetic variations. Other supportive gene–gene and gene–environment studies are also recommended to expand the progress in this field.

## 5. Conclusions

The present findings confirmed the essential contribution of the studied oxidative-stress-related gene variants to obesity risk in the present population. This could help in the risk stratification of obese individuals in this region with other genetic and environmental determinants. Additionally, their encoded antioxidant enzymes could be promising targets for preventive and future individualized therapeutic strategies.

## Figures and Tables

**Table 1:** Clinical and biochemical characteristics of the study population.

Variables	Levels	Non-Obese	Obese	*p*-Value
**Demographic Data**				
Age, years	Mean ± SD	36.96 ± 14.7	41.49 ± 16.2	0.005 *
Sex	Female	78 (33.9)	65 (42.2)	0.10
	Male	152 (66.1)	89 (57.8)	
BMI, kg/m^2^	Mean ± SD	21.36 ± 3.2	33.65 ± 3.9	<0.001 *
**Comorbidities**				
T2DM	Positive	99 (43.0)	78 (50.6)	0.14
Dyslipidemia	Positive	26 (11.3)	35 (22.7)	0.004 *
Hypertension	Positive	27 (11.7)	31 (20.1)	0.029 *
	SBP, mmHg	123.62 ± 18.0	126.14 ± 18.6	0.18
	DBP, mmHg	72.5 ± 9.95	72.11 ± 9.60	0.65
**Laboratory Data**				
Glycemic state	Serum glucose, mmol/L	5.7 (4.7–6.9)	6.0 (5.24–7.7)	0.003 *
	Glycosylated hemoglobin (HbA1c), %	6.0 ± 1.67	6.27 ± 1.77	0.13
	Serum insulin, mIU/L	160 (73.1–324.5)	220 (86–448)	0.006 *
Lipid profile	Triglyceride, mmol/L	1.51 (1.03–2.3)	1.58 (1.04–2.5)	0.80
	Total cholesterol, mmol/L	4.83 ± 0.99	4.85 ± 1.07	0.85
	HDL-cholesterol, mmol/L	1.16 (0.97–1.35)	1.14 (0.97–1.33)	0.61
	LDL-cholesterol, mmol/L	2.73 (2.24–3.53)	2.91 (2.19–3.5)	0.74

Data are shown as number (percentage), mean ± standard deviation (SD), or median (interquartile range). Two-sided Chi-square, Fisher’s exact, Student’s t, and Mann–Whitney U tests were used. (*) Indicates significance at *p*-value < 0.05. Abbreviations; T2DM: type 2 diabetes mellitus, SBP: systolic blood pressure, DBP: diastolic blood pressure, HDL: high-density lipoprotein, LDL: low-density lipoprotein.

**Table 2:** Association of gene variant panel with the risk of obesity.

Gene	SNP ID	Alleles	Non-Obese	Obese	Genotypes	Non-Obese	Obese	Model	Adjusted OR (95%CI)	*p*-Value
*GSTM1 *	rs1056806	**C allele**	416 (93)	270 (88)	**C/C**	195 (87)	119 (78)	**Heterozygote**	2.02 (1.15–3.55)	0.015 *
		**T allele**	32 (7)	36 (12)	**C/T**	26 (12)	32 (21)	**Homozygote**	1.09 (0.18–6.63)	0.05
		***p*-value**	0.22		**T/T**	3 (1)	2 (1)	**Dominant**	1.92 (1.11–3.31)	0.019 *
					***p*-value**	0.039 *		**Recessive**	0.98 (0.16–5.91)	0.98
*GSTT1 *	rs1111875	**A allele**	259 (76)	159 (75)	**A/A**	96 (56)	61 (58)	**Heterozygote**	0.87 (0.52–1.45)	0.42
		**C allele**	81 (24)	53 (25)	**A/C**	67 (39)	37 (35)	**Homozygote**	1.80 (0.62–5.21)	0.45
		***p*-value**	0.86		**C/C**	7 (4)	8 (8)	**Dominant**	0.96 (0.59–1.56)	0.86
					***p*-value**	0.41		**Recessive**	1.90 (0.67–5.40)	0.23
*GSTP1 *	rs1695	**A allele**	221 (65)	133 (62)	**A/A**	73 (43)	45 (42	**Heterozygote**	0.93 (0.55–1.58)	0.46
		**G allele**	117 (35)	81 (38)	**A/G**	75 (44)	43 (40)	**Homozygote**	1.47 (0.71–3.03)	0.49
		***p*-value**	0.65		**G/G**	21 (12)	19 (18)	**Dominant**	1.05 (0.64–1.71)	0.85
					***p*-value**	0.45		**Recessive**	1.52 (0.78–2.99)	0.22
*MGST3 *	rs7744724	**C allele**	339 (96)	214 (98)	**C/C**	165 (93)	105 (96)	**Heterozygote**	0.70 (0.21–2.33)	0.20
		**T allele**	15 (4)	4 (2)	**C/T**	9 (5)	4 (4)	**Homozygote**	0.00 (0.00–NA)	0.57
		***p*-value**	0.11		**T/T**	3 (2)	0 (0)	**Dominant**	0.52 (0.16–1.67)	0.25
					***p*-value**	0.33		**Recessive**	0.00 (0.00–NA)	0.09
*SOD1 *	rs2234694	**A allele**	338 (97)	209 (100)	**A/A**	166 (95)	104 (99)	**Heterozygote**	0.27 (0.03–2.24)	0.15
		**C allele**	10 (3)	1 (0)	**A/C**	6 (3)	1 (1)	**Homozygote**	0.00 (0.00–NA)	0.17
		***p*-value**	0.048 *		**C/C**	2 (1)	0 (0)	**Dominant**	0.20 (0.02–1.62)	0.07
					***p*-value**	0.23		**Recessive**	0.00 (0.00–NA)	0.17
*SOD2 *	rs4880	**A allele**	191 (57)	102 (48)	**A/A**	58 (35)	26 (25)	**Heterozygote**	1.49 (0.83–2.67)	0.13
		**G allele**	143 (43)	110 (52)	**A/G**	75 (45)	50 (47)	**Homozygote**	1.97 (1.00–3.86)	0.045 *
		***p*-value**	0.038 *		**G/G**	34 (20)	30 (28)	**Dominant**	1.64 (0.95–2.82)	0.07
					***p*-value**	0.13		**Recessive**	1.54 (0.88–2.72)	0.13
*SOD3 *	rs2536512	**A allele**	170 (51)	118 (56)	**A/A**	39 (23)	38 (36)	**Heterozygote**	0.47 (0.26–0.83)	0.033 *
		**G allele**	166 (49)	92 (44)	**A/G**	92 (55)	42 (40)	**Homozygote**	0.69 (0.35–1.36)	0.07
		***p*-value**	<0.001 *		**G/G**	37 (22)	25 (24)	**Dominant**	0.53 (0.31–0.91)	0.021 *
					***p*-value**	0.033 *		**Recessive**	1.11 (0.62–1.97)	0.73
*CAT*	rs7943316	**A allele**	116 (47)	52 (40)	**A/A**	34 (28)	13 (20)	**Heterozygote**	0.85 (0.43–1.69)	0.46
		**T allele**	130 (53)	78 (60)	**T/A**	48 (39)	26 (40)	**Homozygote**	0.60 (0.27–1.35)	0.90
		***p*-value**	0.18		**T/T**	41 (33)	26 (40)	**Dominant**	0.75 (0.40–1.40)	0.37
					***p*-value**	0.46		**Recessive**	0.65 (0.32–1.35)	0.24
*GPX1 *	rs1800668	**A allele**	83 (19)	72 (25)	**A/A**	9 (4)	15 (10)	**Heterozygote**	1.03 (0.64–1.65)	0.77
		**G allele**	345 (81)	218 (75)	**G/A**	65 (30)	42 (29)	**Homozygote**	2.65 (1.11–6.32)	0.048 *
		***p*-value**	0.041 * (M-H)		**G/G**	140 (65)	88 (61)	**Dominant**	1.23 (0.79–1.90)	0.36
					***p*-value**	0.07		**Recessive**	2.63 (1.12–6.18)	0.024 *
*GPX4 *	rs713041	**C allele**	176 (52)	111 (53)	**C/C**	42 (25)	27 (26)	**Heterozygote**	0.96 (0.54–1.73)	0.97
		**T allele**	160 (48)	97 (47)	**C/T**	92 (55)	57 (55)	**Homozygote**	0.92 (0.44–1.91)	0.99
		***p*-value**	0.82		**T/T**	34 (20)	20 (19)	**Dominant**	0.95 (0.54–1.67)	0.86
					***p*-value**	0.48		**Recessive**	0.94 (0.51–1.74)	0.84
*NOS3 *	rs1799983	**G allele**	254 (75)	176 (83)	**G/G**	97 (57)	73 (69)	**Heterozygote**	0.66 (0.39–1.13)	0.09
		**T allele**	84 (25)	36 (17)	**T/G**	60 (36)	30 (28)	**Homozygote**	0.33 (0.09–1.22)	0.21
		***p*-value**	0.029 *		**T/T**	12 (7)	3 (3)	**Dominant**	0.61 (0.37–1.02)	0.06
					***p*-value**	0.10		**Recessive**	0.38 (0.10–1.38)	0.11
*NOS2*	rs2297518	**A allele**	123 (36)	82 (38)	**A/A**	23 (13)	13 (12)	**Heterozygote**	1.34 (0.80–2.26)	0.51
		**G allele**	221 (64)	134 (62)	**A/G**	77 (45)	56 (52)	**Homozygote**	1.04 (0.48-2.29)	0.25
		***p*-value**	0.59		**G/G**	72 (42)	39 (36)	**Dominant**	1.27 (0.78-2.09)	0.34
					***p*-value**	0.51		**Recessive**	0.89 (0.43-1.83)	0.74

Binary regression analysis was performed to estimate the adjusted risk of obesity in each genotype’s presence according to various genetic association models. The odds ratio (OR) and 95% confidence interval (CI) for each model are shown. The adjustment was performed by age and sex. MH: Mantel–Haenszel chi-square test. (*) Indicates significance at *p*-value < 0.05.

**Table 3:** Genotype combination analysis of risk alleles for obesity.

No.	*GSTM1*	*SOD2*	*SOD3*	*GPX1*	Frequency	OR (95% CI)	*p*-Value
1	C	A	A	G	0.126	1	---
2	C	A	G	G	0.117	3.05 (0.66–14.14)	0.15
3	C	G	A	G	0.191	2.95 (1.27–6.86)	0.012 *
4	C	G	G	G	0.111	2.06 (0.35–11.94)	0.42
5	C	G	A	G	0.080	6.15 (1.46–25.87)	0.014 *
6	C	A	A	G	0.067	0.52 (0.03–8.27)	0.64
7	C	A	G	G	0.064	0.74 (0.10–5.58)	0.77
8	C	A	A	A	0.043	5.00 (1.23–20.33)	0.025 *
9	C	G	G	G	0.039	1.85 (0.02–209.77)	0.80
10	T	G	G	G	0.030	3.25 (1.06–9.95)	0.040 *
11	C	G	A	A	0.026	1.45 (0.16–13.23)	0.74
12	C	A	A	A	0.021	9.44 (0.73–122.24)	0.09
13	T	A	A	G	0.019	1.85 (0.02–209.77)	0.80

Combinations with frequencies < 0.01 were excluded. (*) Indicates significance at *p*-value < 0.05.

## Data Availability

All generated data in this study are included in the article and supplementary material.

## Supplementary Materials

The following is available online at https://www.mdpi.com/article/10.3390/antiox10040595/s1, Table S1: The ready-made assay probe sequences applied in the present study.
