# Supplementary material for: Antioxidants-Related Superoxide Dismutase (SOD), Catalase (CAT), Glutathione Peroxidase (GPX), Glutathione-S-Transferase (GST), and Nitric Oxide Synthase (NOS) Gene Variants Analysis in an Obese Population: A Preliminary Case-Control Study
Source: Antioxidants (Basel). 2021 Apr 13;10(4):595. doi: 10.3390/antiox10040595 (PMC8070436; doi:10.3390/antiox10040595)
Supplement: Supplementary file 1 [file antioxidants-10-00595-s001.pdf]

Table S1: The ready-made assay probe sequences applied in the present study

Genes	No	polymorphism	Probe sequence	Reference sequence (rs) Assay ID
GST	1	GSTM1 [C/T]	GTATATTTGAGCCCAAGTGCTTGGA[C/T]GCCTTCCCAAATCTGAAGGACTTCA	rs1056806 C_175674977_10
	2	GSTT1 [A/C]	CTTCTTGAGGGGGTTCACCTGGGCA[A/C]AGGCATCGCTTAAGTGCTGACCTGG	rs17856199 C__11214581_10
	3	GSTP1 [A/G]	CGTGGAGGACCTCCGCTGCAAATAC[A/G]TCTCCCTCATCTACACCAACTATGT	rs1695 C___3237198_20
	4	MGST3 [C/T]	GACCCTGAGTGCAGGTTCAGACGTC[C/T]AGAGGAAATGACTTGATGGTACGGA	rs2065942 C__11214581_10
SOD	5	SOD1 [A/C]	ATGCTTAACTCTTGTAATAATGGCG[A/C]TAGCTTTCTGGAGTTCATATGGTAT	rs2234694 C__34770911_10
	6	SOD2 [A/G]	CTGCCTGGAGCCCAGATACCCCAAA[A/G]CCGGAGCCAGCTGCCTGCTGGTGCT	rs4880 C___8709053_10
	7	SOD3 [A/G]	CATGCAGCGGCGGGACGACGACGGC[A/G]CGCTCCACGCCGCCTGCCAGGTGCA	rs2536512 C___2668728_10
CAT	8	CAT [A/T]	TTGGCTGAGCCTGAAGTCGCCACGG[A/T]CTCGGGGCAACAGGCAGATTTGCCT	rs7943316 C___1883210_10
GPX	9	GPX1 [G/A]	CAGCGGAGCGCCCCGAACAAGCACT[G/A]TAAGGGGAGGCCAGCAGGCGCCTCC	rs1800668 C___7912052_40
	10	GPX4 [C/T]	CCGCCCGAGCCCCTGCCCACGCCCT[C/T]GGAGCCTTCCACCGGCACTCATGAC	rs713041 C___2561693_20
NOS	11	nNOS/NOS2 [A/G]	GTTGAGCTCTTTCAGCATGAAGAGC[A/G]ATTTCTTCAGTTTCTAGAAAGAGAG	rs2297518 C__11889257_10
	12	eNOS/NOS3 [G/T]	CCCTGCTGCTGCAGGCCCCAGATGA[G/T]CCCCCAGAACTCTTCCTTCTGCCCC	rs1799983 C___3219460_20

Data source: Thermo Fisher Scientific, Applied Biosystems, USA.
